# Right and left ventricular function and myocardial scarring in adult patients with sickle cell disease: a comprehensive magnetic resonance assessment of hepatic and myocardial iron overload

**DOI:** 10.1186/1532-429X-15-83

**Published:** 2013-09-19

**Authors:** Flávia P Junqueira, Juliano L Fernandes, Guilherme M Cunha, Tadeu T A Kubo, Claudio M A O Lima, Daniel B P Lima, Marly Uellendhal, Sidney R Sales, Carolina A S Cunha, Viviani L R de Pessoa, Clarisse L C Lobo, Edson Marchiori

**Affiliations:** 1 Clínica de Diagnóstico Por Imagem (CDPI), Av. das Américas 4666 sala 325, Barra da Tijuca, Rio de Janeiro 22649-900RJ, Brazil; 2 Department of Radiology, Federal University of Rio de Janeiro, Rio de Janeiro, Brazil; 3 Universidade Estadual de Campinas, Campinas, Brazil; 4 Delboni Auriemo Medicina Diagnóstica, São Paulo, Brazil; 5 HEMORIO, Rio de Janeiro, Brazil

## Abstract

**Background:**

Patients with Sickle cell disease (SCD) who receive regular transfusions are at risk for developing cardiac toxicity from iron overload. The aim of this study was to assess right and left cardiac volumes and function, late gadolinium enhancement (LGE) and iron deposits in patients with SCD using CMR, correlating these values with transfusion burden, ferritin and hemoglobin levels.

**Methods:**

Thirty patients with SCD older than 20 years of age were studied in a 1.5 T scanner and compared to age- and sex-matched normal controls. Patients underwent analysis of biventricular volumes and function, LGE and T2* assessment of the liver and heart.

**Results:**

When compared to controls, patients with SCD presented higher left ventricular (LV) volumes with decreased ejection fraction (EF) with an increase in stroke volume (SV) and LV hypertrophy. The right ventricle (RV) also presented with a decreased EF and hypertrophy, with an increased end-systolic volume. Although twenty-six patients had increased liver iron concentrations (median liver iron concentration value was 11.83 ± 9.66 mg/g), only one patient demonstrated an abnormal heart T2* < 20 msec. Only four patients (13%) LGE, with only one patient with an ischemic pattern.

**Conclusions:**

Abnormal heart iron levels and myocardial scars are not a common finding in SCD despite increased liver iron overload. The significantly different ventricular function seen in SCD compared to normal suggests the changes in RV and LV function may not be due to the anemia alone. Future studies are necessary to confirm this association.

## Background

Sickle cell disease (SCD) is an inherited hemoglobin synthesis disorder characterized by life-long severe hemolytic anemia, pain crises, and chronic organ damage [1]. Patients with SCD who receive regular transfusions are at risk of cellular toxicity and cardiac failure due to iron overload. Although many studies have demonstrated risk factors for liver iron overload in these patients, few have examined cardiac iron deposition [2,3].

Cardiovascular magnetic resonance (CMR) is a useful noninvasive tool for evaluating the amount of iron in the heart. The technique relies on the measurement of T2 star (T2*) relaxation from gradient-echo sequences. When the storage capacity of ferritin is exceeded, iron is deposited in the myocardium as particulate hemosiderin, a form of ferrihydrite (hydrated iron oxide). This deposition disrupts local magnetic field homogeneity, reducing T2* values in an inverse relationship to iron concentration [4]. T2* CMR is an ideal technique for the noninvasive measurement of iron concentration because image acquisition using the validated single-slice method requires only a single breath hold and has good reproducibility, making it valuable for serial monitoring over time. Calibration of the T2* technique in humans has been reported [5-7]. CMR is also considered to be the most accurate and reproducible technique for assessing right ventricle (RV) and left ventricle (LV) volumes and ejection fraction (EF) [8].

The complications of SCD are multiple. The two most common acute events are vaso-occlusive pain crisis and acute chest syndrome (ACS).The vaso-occlusive events of SCD may occur in any organ, including the heart and lungs. Additionally, patients are at risk for a progressive vasculopathy characterized by systemic and pulmonary hypertension (PH), endothelial dysfunction, and proliferative changes in the intima and smooth muscle of blood vessels. With increasing age, the incidence of chronic end-organ complications, including chronic renal failure, osteonecrosis, and PH, increases. The pulmonary complications of SCD are of particular importance, as ACS and PH have the highest associated mortality rates within this population [9]. Thus, not only can cardiac function indices in these patients differ from those in the normal population, but vaso-occlusive crisis can also affect the heart. Despite the high incidence of these complications, their exact mechanisms and effects on the heart are not fully understood because myocardial infarctions are infrequently reported. Echocardiography has many limitations in this context, especially in the evaluation of PH and RV assessment [10].

Given that CMR can provide a comprehensive and unique evaluation of the heart in a single examination, the aims of this study were to assess the right and left cardiac volumes and function, late gadolinium enhancement (LGE), and iron deposits in patients with SCD aged > 20 years, and to correlate these values with transfusion burden and ferritin and hemoglobin levels.

## Methods

### Study population

This single-site, prospective, clinical observational study involved 30 consecutive patients (10 males and 20 females) with SCD who were referred for initial myocardial T2* examination from a specialized hematology center (Hemorio, Rio de Janeiro, Brazil). The patients’ mean age was 37.5 ± 14.8 years. Twenty-seven patients were receiving partial exchange transfusions (at least one per month). Transfusion burden was defined as >20 red blood cell transfusion events (from initial transfusion). Red blood cell transfusion in sickle cell was indicated only in situations of acute losses, or when the hemoglobin fell two or more grams below its baseline level. Seven percent of the patients were on transfusions for past history of cerebrovascular accidents, 2% because of osseous infarction and 1% because of perimaleolar ulcers (Table 1).

**Table 1:** Patient demographics, hematological and clinical profiles, medication use, and CMR T2 star (*) parameters

**Patient demographics**	
Total number of patients	30
Age (years)	37.5 ± 14.8
Height (cm)	163 ± 0.1
Weight (Kg)	60.3 ± 9.1
**Haematological profile**	
Serum ferritin (mg/L)	2787.9 ± 1857
Hemoglobin (g/dL)	8.89 ± 1.57
**Clinical profile n (%)**	
Cerebrovascular accident	7 (23.3%)
Perimaleolar ulcers	1 (3.3%)
Osseous infarcts	2 (6.6%)
**Medication use n (%)**	
Folic Acid	4 (13.3%)
Hidroxyurea	4 (13.3%)
Deferoxamine	2 (6.6%)
Deferasirox	4 (13.3%)
CMR T2* parameters	
Cardiac T2* (ms)	37.6 ± 7.1
Liver T2* (ms)	4.48 ± 4.39
Liver Iron Concentration (mg/g)	11.83 ± 9.66

Six patients were being treated with a single iron chelation agent (deferoxamine or deferasirox) at presentation, and the other patients were receiving folic acid and hydroxyurea. Only patients on a regular transfusion regiment were included in the final analysis. The Hemorio Research Ethics Committee approved the data collection and analysis associated with this study, and all patients signed informed consent forms.

### Magnetic resonance imaging

All patients underwent magnetic resonance using a 1.5T Avanto scanner (Siemens Medical Systems, Erlangen, Germany) operating at a maximum gradient strength of 45 mT m^–1^ and slew rate of 200 T m^–1^ s^–1^. Each scan (duration, 20–30 min) included the measurement of hepatic (right lobe) and myocardial (mid-septum) T2*, LV and RV volumes, EF, mass, and LGE using previously published techniques [11-13].

For the measurement of hepatic T2*, a single transverse slice was acquired using a single breath-hold electrocardiography (ECG)-gated multi-echo technique [repetition time (TR): 200 ms, slice thickness: 10 mm, flip angle: 20°, field of view (FOV): 400 × 238 mm, matrix: 128 × 76 mm, bandwidth: 1955 Hz/Px]. This T2* sequence generated a series of 12 images with echo times (TEs) of 1.0–16.5 ms and echo spacing of 1.4 ms.

For the measurement of myocardial T2*, a single short-axis midventricular slice was acquired using a single breath-hold ECG-gated multi-echo dark blood technique (TR: 710 ms, slice thickness: 10 mm, flip angle: 20°, FOV: 400 × 300 mm, matrix: 256 × 96 mm, bandwidth: 810 Hz/Px). This T2* sequence generated a series of eight images with TEs of 1.9–17.3 ms and spacing of 1.7 ms.

For LV and RV functional evaluation, the protocol included a breath-hold ECG-gated cine true fast imaging with steady-state precession (FISP) sequence in the short axis plane (TR: 50.4 ms, TE: 1.2 ms, FOV: 280 × 228 mm, matrix: 192 × 117 mm; flip angle: 15°; slice thickness: 6.5 mm, number of slices: 12). In addition, 10 min after intravenous administration of 0.2 mmol kg^–1^ gadodiamide (Dotaren^TM^; Guerbet, Villepinte, France), a single-shot breath-hold ECG-gated phase-sensitive inversion recovery sequence was acquired in the short, horizontal and vertical long axis planes (TR: 555 ms, TE: 1.1 ms, inversion time: 250 ms, FOV: 340 × 255 mm; matrix: 192 × 116 mm; flip angle: 25°, slice thickness: 7 mm, bandwidth: 1445 Hz/Px) using the same position and number of slices as the cine true FISP sequence, to evaluate LGE.

### Image analysis

Two experienced radiologists analyzed the MR images at a dedicated workstation (Leonardo; Siemens Medical Systems) using ARGUS and Viewer software (Siemens Medical Systems). For hepatic T2* analysis, regions of interest (ROIs) were manually drawn in the liver using Viewing software while avoiding all major visible vessels. For myocardial T2* analysis, a full-thickness ROI was manually defined in the interventricular septum (routinely chosen to avoid T2* artifacts from the cardiac veins, liver, and lungs).

Hepatic and myocardial T2* decay were calculated using manual analysis in an electronic spreadsheet [14]. Signal intensity was plotted against TE for each image, and T2* was calculated from the resulting exponential decay curve. A truncation method was used to allow for background noise, as previously described [15]. Liver iron concentration (LIC) was calculated using the calibrated technique published by Hankins et al. [16]. We used a previously published normal range and lower cutoff value for myocardial T2* obtained from a series of healthy volunteers (median normal value: 40 ms, lower cutoff value for normality: 20 ms) [17]. This cutoff value is widely accepted in clinical practice. RV and LV volumes were determined from steady-state free-precession cines, with contiguous short-axis slices from base to apex, as previously described [12,13]. Ventricular volumes and EF were analyzed with ARGUS software. RV and LV endocardial and epicardial borders were delineated in all phases of the cardiac cycle on short-axis slices. The papillary muscles and RV trabeculations were excluded from ventricular volume measurements. LV and RV volumes were indexed to body surface area [12,13].

The normal ranges for LV and RV volumes and function were taken from normal, non-anemic adult Latin American patients of the same sexes and ages [18], with the lower limit of normal for RV EF and LV in healthy subjects being 54% and 56% respectively [12,13].

### Statistical analysis

All parameters are presented as means ± standard deviations. Spearman’s rank test was used to assess correlations among hepatic and myocardial T2*, blood transfusions, hemoglobin and ferritin levels, RV and LV volumes, EF, and LGE. Analysis of variance was used to assess differences across different ranges of hepatic and myocardial T2*. Two-sided statistical significance was set at p < 0.05. All statistical analyses were performed using R software (ver. 2.15.1; Vanderbilt University, Nashville TN, USA).

## Results

The clinical and hematological parameters of patients with SCD, and CMR parameters of control subjects and patients with SCD, are shown in Tables 1 and 2. Serum ferritin levels ranged from 1045 to 6398 mg/dL (mean: 2787.9 ± 1857 mg/dL). Median hepatic and myocardial T2* values in this population were 4.48 ± 4.39 ms and 37.6 ± 7.1 ms, respectively. LV EF was 62.5 ± 4.54% and RV EF was 50.3 ± 7.6%. Median LIC was 11.83 ± 9.66 mg/g.

**Table 2:** CMR parameters: RV and LV function

**CMR parameters**	**Controls (n = 30)**	**SCD patients (n = 27)**	**P value**
**Left ventricle**			
Ejection Fraction (%)	66.5 ± 7.2	62.5 ± 4.54	0.017
End-diastolic volume index (mL/m2)	70.8 ± 14.8	96.2 ± 16.5	<0.001
End-systolic volume index (mL/m2)	23.8 ± 7.9	36.5 ± 8.9	<0.001
Stroke volume index (mL/m2)	47.0 ± 8.8	59.7 ± 9.1	<0.001
Mass index (mL/m2)	50.1 ± 12.2	78.5 ± 12.7	<0.001
**Right ventricle**			
Ejection Fraction (%)	60.4 ± 7.2	50.3 ± 7.6	<0.001
End-diastolic volume index (mL/m2)	73.8 ±13.9	70.8 ±17.8	0.481
End-systolic volume index (mL/m2)	29.2 ± 8.4	35.7 ± 12.9	0.027
Stroke volume index (mL/m2)	44.7 ± 8.5	35.1 ± 7.7	<0.001
Mass index (mL/m2)	16.5 ± 2.4	23.8 ± 5.4	<0.001
**Positive LGE**	0%	13.30%	

Compared with control subjects, patients with SCD in this study had decreased LV EF (p = 0.017) and LV dilatation in diastole and systole (both p <0.001), leading to an increased stroke volume (SV) with LV hypertrophy (p < 0.001; Table 2). Decreased RV EF and SV (both p < 0.001) and increased end-systolic volume (ESV; p = 0.027) and RV hypertrophy (p < 0.001) were also observed. However, no RV dilatation in diastole (p = 0.481) in comparison with normal reference values was present (Table 2).

**Figure 1:**
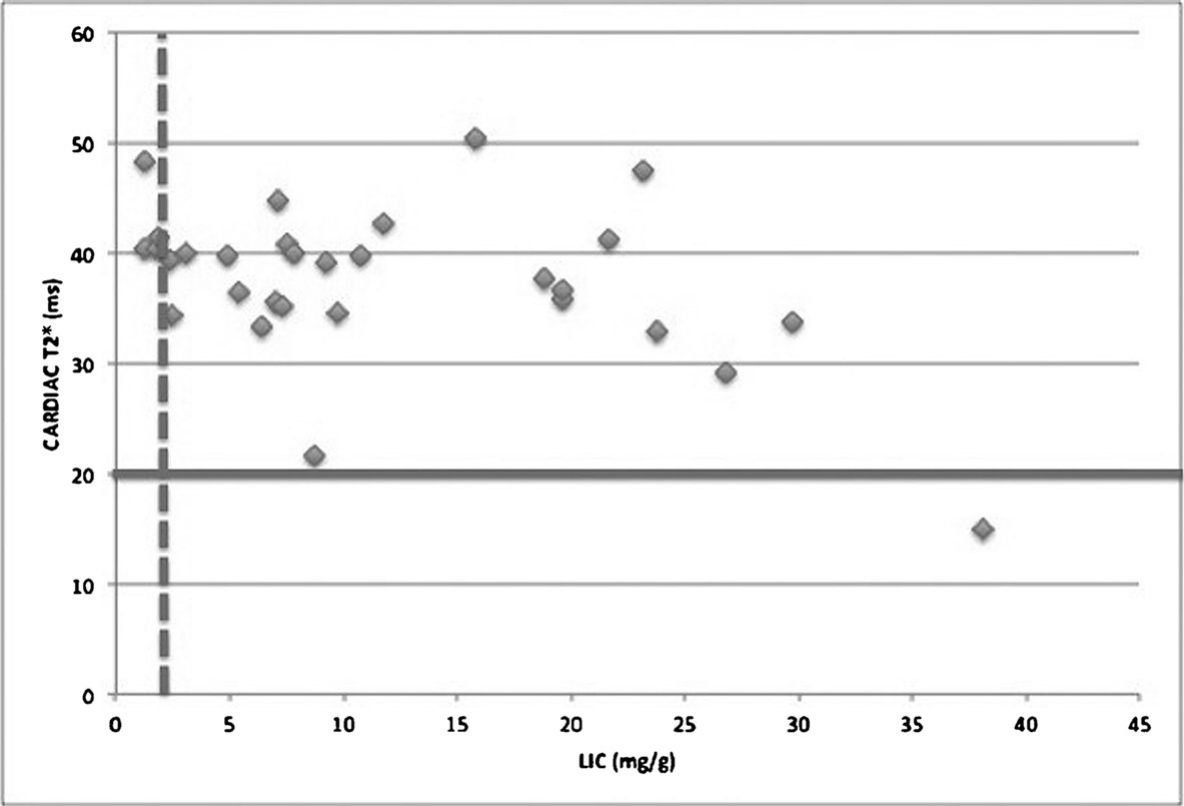
Relationship between LIC and myocardial T2* values. The vertical broken line shows the upper limit of the normal range for and LIC of 2.0 mg/g. The horizontal line shows the lower limit of the normal range for a myocardial T2* of 20 ms [16,17].

Twenty-six (86.7%) patients had abnormal LICs. Although hepatic iron overload was severe, abnormal (15 ms) and borderline low (21.7 ms) myocardial T2* values were found in only one (3.3%) patient each (Figure 1). there was a significant correlation between RV and LV EDV. Correlations among serum ferritin and hemoglobin levels, transfusion burden, myocardial and hepatic T2* values, RV and LV EF, end-diastolic volume (EDV), ESV, and LGE are shown in Table 3.

**Table 3:** Correlations among ferritin and hemoglobin levels, transfusion burden, myocardial and hepatic T2* values, RV and LV EF and EDV, and LGE

**Correlation**	**R value**	**P value**
Liver T2* x Transfusion burden	0.425	0.019
Liver T2* x Ferritin	−0.469	0.010
Transfusion burden x Cardiac T2*	−0.216	0.251
Transfusion burden x Ferritin	−0.150	0.436
Transfusion burden x LV EDV	−0.129	0.496
Transfusion burden x RV EDV	0.065	0.731
Transfusion burden X LV EF	0.076	0.765
Transfusion burden X RV EF	−0.449	0.062
Cardiac T2* x RV EDV	−0.023	0.906
Cardiac T2* x RV EF	−0.219	0.245
Cardiac T2* x LV EDV	0.012	0.949
Cardiac T2* x LV EF	−0.228	0.225
Cardiac T2* x Liver T2*	0.300	0.107
Cardiac T2* x Ferritin	−0.353	0.061
RV EF x Liver T2*	0.065	0.732
LV EF x Liver T2*	−0.155	0.414
RV EF x Hemoglobin levels	0.066	0.732
LV EF x Hemoglobin levels	0.301	0.112
RV EF x Transfusion burden	−0.275	0.732
LV EF x Transfusion burden	−0.022	0.141
RV EF x Ferritin	−0.042	0.828
LV EF x Ferritin	0.131	0.497
RV EF x LV EF	0.356	0.053
RV EDV x LV EDV	0.447	0.013
Cardiac T2* x LGE	−0.140	0.476
RV EDV x LGE	0.069	0.727
LV EDV x LGE	0.089	0.652

**Figure 2:**
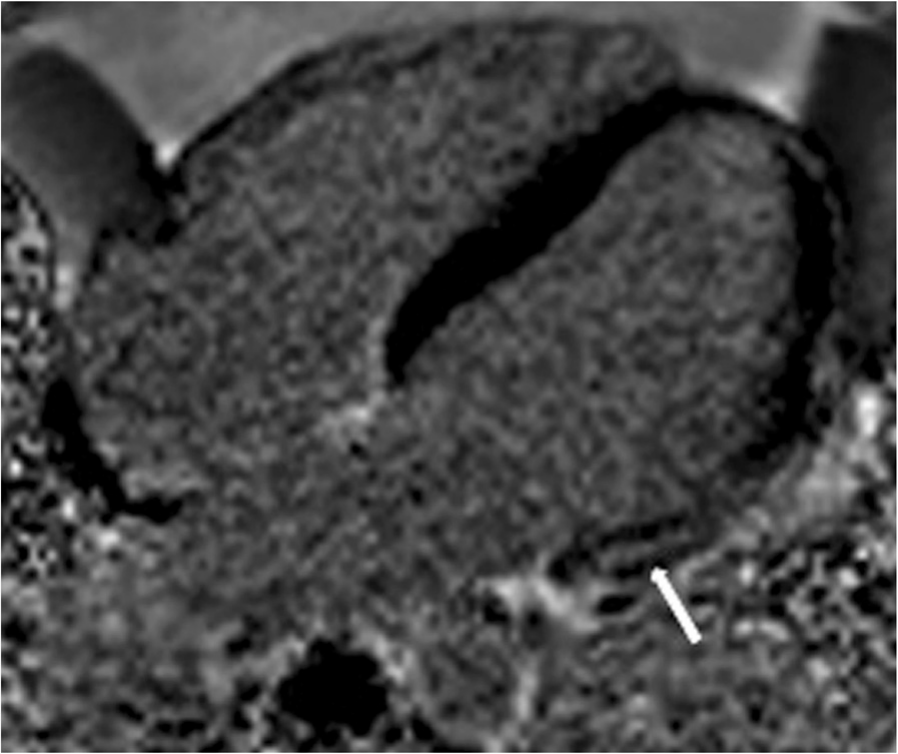
**Example of myocardial delayed enhancement.** Horizontal long-axis phase-sensitive inversion recovery images of one patient show myocardial delayed enhancement, demonstrated as a hyperintense mesocardial area in the basal segment of the lateral wall (arrow), with a non-ischemic pattern.

Four (13.3%) patients presented with LGE, with an ischemic pattern (subendocardial or transmural) in one case and non-ischemic patterns (mesocardial or epicardial) in three cases (Figure 2, Table 2). Myocardial T2* values and LV and RV EDV were weakly correlated with LGE (Table 3).

One patient showed signs of pulmonary hypertension with right atrial and RV enlargement, moderate RV dysfunction, displacement of the interventricular septum to the left during end diastole and systole, and small areas of mesocardial LGE at the insertion points of the free RV wall in the interventricular septum. An echocardiogram confirmed that this patient’s mean pulmonary artery pressure was 42 mm Hg.

## Discussion

The importance of the RV as an aggravating factor in heart disease and a predictor of adverse cardiac outcomes has often been overlooked in the past. Our study provides new data showing that the RV can be altered in patients with SCD, a finding not deeply studied in SCD and are worth reporting. Our main findings were a decreased RV EF, RV dilatation in systole, increased SV and RV hypertrophy, and signs of pulmonary hypertension in one patient. CMR plays an important role in the evaluation of the right chambers, especially in situations in which echocardiography is limited [10]. CMR is the gold standard technique for the assessment of ventricular function and the quantification of volume and mass without the incorporation of geometric assumptions. RV volumes, mass, and function can be quantified with excellent intra- and interobserver variability and good interstudy reproducibility [19].

SCD is an important cause of morbidity and mortality worldwide, causing damage and dysfunction in multiple organs, including the heart. Cardiac dilatation, which has been reported previously in patients with SCD, is due in part to the anemia underlying this disease but may be compounded by other factors, possibly leading to unique cardiomyopathy [20]. Our LV findings also confirm those of previous studies [20-22], excepting decreased EF. In this study, patients with SCD had decreased LV EF, LV dilatation in diastole and systole, and increased SV with LV hypertrophy compared with normal individuals [18].

In this study, we used CMR to evaluate myocardial iron loading and regional fibrosis using the LGE technique, two factors that may explain the presence of cardiomyopathy in patients with SCD. LGE is related to myocyte damage and can be observed in scar, focal or diffuse fibrosis, necrosis, inflammatory processes and tumors. The damaged myocardium retains the contrast for a longer time compared to normal myocardium and hyperintense areas, and can be seen 10–20 minutes after the administration of the contrast media (Gd). In SCD patients much of the abnormal cardiac assessment is not associated with cardiac iron deposition. However fibrosis can occur related to pulmonary hypertension, coronary disease, cardiac dilatation or hypertrophy.

Myocardial iron loading increases ESV and reduces LV EF [17,23], but only one patient in our sample showed slight loading despite a wide range of transfusion burdens, suggesting that significant myocardial iron overload is uncommon in SCD. This finding contrasts with the related transfusional hemoglobinopathy in thalassemia major (TM), in which iron-induced cardiac injury is the leading cause of morbidity and mortality [24]. It implies that the handling of excess transfusional iron in these conditions may differ. The gastrointestinal absorption of excess iron in TM, which may not occur in SCD, may in part explain this phenomenon [25]. However, patients with SCD are significantly less iron loaded than most patients with TM, and a threshold level may define the point at which significant myocardial iron loading occurs [26]. Evidence of focal fibrosis was present in four patients in our sample. The single patient with an ischemic pattern exhibited LV enlargement with normal global function and segmental akinesia of the apical inferior wall of the LV, associated with transmural LGE. One patient with a non-ischemic pattern presented right atrial and RV enlargement, moderate RV dysfunction, and small areas of mesocardial LGE at the insertion points of the free RV wall in the interventricular septum. Another patient presented discrete LV and RV enlargement, and mesocardial LGE at the insertion points of the free RV wall in the interventricular septum and basal and medial segments of the septal inferior wall, with no related regional wall motion abnormality. Another patient with a non-ischemic pattern presented mesocardial LGE at the basal segment of the lateral LV wall, with no related regional wall motion abnormality and normal LV volumes and function. These findings support the idea that occult regional fibrosis may occur, as Westwood et al. [3] reported previously, although the incidence of this condition is low (13% of our cases and 4% in their series).

Possible explanations for this low incidence may be that the areas of fibrosis resulting from microvascular sludge infarct may be too small to detect. More importantly, perhaps, is the reliance of LGE on the contrast between areas of normal and abnormal myocardium. A diffuse fibrotic abnormality throughout the myocardium would be difficult to assess. Sickle cell infarction may be a more diffuse process affecting the whole myocardium, and T1 mapping techniques may allow more accurate assessment.

### Limitations

These T2* values apply only at a field strength of 1.5 T, and the relaxation parameters differ at higher field strengths, such as 3 T, which are becoming more widely available for clinical scanning. We measured myocardial T2* values in the interventricular septum and used them to assess global myocardial iron loading. Although this approach provides an indirect measurement of RV iron, direct measurement of T2* in the free RV wall is not robust because the myocardium is very thin, close to the chest wall, and susceptible to artifacts.

## Conclusions

CMR enables accurate assessment of myocardial iron levels and the detection of even very small areas of focal fibrosis using the LGE technique. However, such abnormalities are not common findings in patients with SCD. The significantly different ventricular function seen in patients with SCD compared with truly normal subjects suggests that changes in RV and LV function may not be due to anemia alone. Future studies are necessary to confirm this association.

## Abbreviations

CMR: Cardiovascular magnetic resonance; ECG: Electrocardiography; EDV: End-diastolic volume; EF: Ejection fraction; ESV: End-systolic volume; FISP: Fast imaging with steady-state precession; FOV: Field of view; LIC: Liver iron concentration; LGE: Late gadolinium enhancement; LV: Left ventricle; PH: Pulmonary hypertension; ROI: Region of interest; RV: Right ventricle; SCD: Sickle cell disease; SV: Stroke volume; T: Tesla; TE: Echo time; TM: Thalassemia major; TR: Repetition time; T2*: T2 star.

## Competing interests

FPJ and JLF have received speakers’ honoraria from Novartis and performed advisory board work for Novartis.

## Authors’ contributions

FPJ and JLF participated equally in the study design, data acquisition, and drafting of the manuscript; GMC, CMAOL, MU, DBPL, CASC, CLCL, and VLRP drafted the manuscript; SRS acquired data; TTAK performed the statistical analysis; EM conceived and co-designed the study, and is responsible for the final manuscript. All authors read and approved the final manuscript.
